# Long Non-Coding RNA Analysis of Vitrified Porcine Immature Oocytes During Maturation and Early Parthenogenetic Embryo Development

**DOI:** 10.3390/cells14221808

**Published:** 2025-11-18

**Authors:** De-Cai Xiang, Zhen He, Shi-Qi Pu, De-Meng Mu, Jing Fu, Wen-Juan Chen, Jun-Yu Jiang, Xue-Mei Li, Bao-Yu Jia, Guo-Quan Wu

**Affiliations:** 1 National Regional Genebank (Yunnan) of Livestock and Poultry Genetic Resources, Yunnan Provincial Engineering Laboratory of Animal Genetic Resource Conservation and Germplasm Enhancement, Yunnan Animal Science and Veterinary Institute, Kunming 650224, China; askalm@163.com (D.-C.X.); 18463287165@163.com (Z.H.); psqshiqi@163.com (S.-Q.P.); Mudemeng1219@163.com (D.-M.M.); fj11260027@163.com (J.F.); chenwenjuanly006@163.com (W.-J.C.); 2 Key Laboratory for Porcine Gene Editing and Xenotransplantation in Yunnan Province, College of Veterinary Medicine, Yunnan Agricultural University, Kunming 650201, China; 13987988411@163.com (J.-Y.J.); lxm19187386050@163.com (X.-M.L.)

**Keywords:** vitrification, long non-coding RNAs, oocyte, early embryo, porcine

## Abstract

The preservation of porcine oocytes is critically important for advancing superior breeds and conserving genetic resources in pig production. Vitrification has gained traction as a preferred alternative to slow freezing for porcine oocytes because of its effectiveness in reducing ice crystal formation, yet it can still negatively affect oocyte quality, compromising their in vitro maturation (IVM) and later embryonic development. Long non-coding RNAs (lncRNAs) have proven to be key players in numerous biological processes, such as oocyte growth, maturation, and early embryogenesis. Despite this, the effects of vitrified porcine germinal vesicle (GV) oocytes, particularly regarding IVM and the dynamic expression patterns of lncRNAs during embryonic development, remain largely unclear. To address this gap, this study conducted lncRNA sequencing at the metaphase II (MII), parthenogenetic 4-cell embryo, and parthenogenetic blastocyst stages sourced from both fresh and vitrified GV oocytes. This method enabled us to ascertain the impact of vitrification on lncRNA expression throughout oocyte maturation and embryonic development. Results identified 773 differentially expressed lncRNAs (DELs) at the MII stage, 1973 at the parthenogenetic 4-cell, and 1192 at the parthenogenetic blastocyst. Enrichment analysis of forecasted target genes revealed their involvement in key regulatory pathways associated with the cell cycle, meiosis, stress response, and metabolic activity. Overall, this study provides a comprehensive overview of lncRNA expression during oocyte maturation and embryonic development following porcine GV oocyte vitrification, thereby shedding light on the molecular mechanisms behind vitrification-induced damage.

## 1. Introduction

In its capacity as a vital livestock species across the globe, the preservation of pig germplasm resources holds immense value for agricultural breeding, biomedical research, and endangered species conservation. Oocyte cryopreservation represents a crucial step in reproductive biotechnology, with vitrification widely adopted because of its effectiveness in reducing ice crystal formation. However, porcine oocytes stand out for their elevated lipid levels, which makes them especially vulnerable to low temperatures [1]. Researchers have successfully vitrified porcine oocytes at both the immature germinal vesicle (GV) and mature metaphase II (MII) stages; they have even proceeded to produce live piglets using these vitrified oocytes [2,3]. Despite these advancements, it remains unclear which stage, immature or mature, offers the most suitable conditions for vitrification. Studies on pig cumulus–oocyte complexes (COCs) have revealed ultrastructural alterations after vitrification, with more pronounced damage at the GV stage, including detachment of cumulus cells from the oocyte, breaches in the zona pellucida, disruption of gap junctions, and diminished mitochondrial matrix density [4]. By contrast, vitrification at the MII stage generally preserves mitochondrial structure and cortical granule distribution, though numerous cytoplasmic vacuoles have been observed [4]. Given the high sensitivity of the MII spindle to temperature fluctuations, immature oocytes may theoretically present advantages for cryopreservation, as they lack a meiotic spindle and still contain the cell nucleus. With continued technological improvements, GV-stage oocytes exhibit relatively high survival rates [5] while maintaining cumulus expansion, nuclear maturation, and fertilization capacity [6,7]. Although blastocysts can be derived from vitrified GV oocytes at appreciable yields [8,9,10], the markedly reduced developmental potential following cryopreservation remains a significant challenge. Previous research has primarily focused on physical cryoinjuries, including membrane rupture, organelle damage, and chromosomal abnormalities [11,12,13,14,15]. However, beyond these physical effects, vitrification also appears to influence gene expression and epigenetic regulation in porcine oocytes. Recent findings demonstrate that stress stimuli during cryopreservation can dysregulate intracellular gene expression, thereby impairing oocyte maturation, fertilization, and subsequent embryonic development [16,17,18]. In addition, epigenetic processes—including DNA methylation, histone alterations, and long non-coding RNAs (lncRNAs)—play pivotal roles in embryogenesis [19,20,21], and disruption of these networks by vitrification may further compromise developmental potential.

LncRNAs constitute RNA sequences greater than 200 nucleotides that lack protein-coding potential [22,23]. Despite not translating into proteins, these molecules are key players in managing how genes express themselves, utilizing various strategies. Cutting-edge research has shown that lncRNAs are essential in the process of ovum maturation as well as the advancement of embryo development. During oocyte maturation, lncRNAs influence gene regulatory networks by interacting with mRNAs, proteins, and other RNAs, thereby affecting the maturation process [24,25,26]. For example, a novel lncRNA with protein-binding properties was identified in buffalo oocytes, suggesting a regulatory role in oocyte maturation [27]. Similarly, MSTRG.17927 regulates the phosphatidylinositol 3-kinase (PI3K) signaling pathway during sheep oocyte maturation [25]. In embryonic development, lncRNAs are involved in essential processes such as maternal-to-zygotic transition (MZT), zygotic genome activation (ZGA), cell fate specification, and organogenesis [28,29]. For instance, lncFKBPL functions as an enhancer lncRNA that regulates ZGA in pigs and supports early embryonic development [30], while the expression of lncRNA 3720 has been shown to influence goats’ early embryonic development [31]. The MZT in pig embryos initiates later, primarily occurring during the 4-cell stage [32]. At this stage, the genetic information required for embryonic development gradually shifts from maternal RNA/proteins stored in the oocyte to embryo-specific genome activation (EGA) [33]. The blastocyst represents a critical developmental milestone, marking the transition from cell division to cell differentiation [34]. Therefore, sequencing analysis of porcine MII oocytes, 4-cell embryos, and blastocysts is essential for comprehensively evaluating the maturity and developmental potential of vitrified GV pig oocytes.

While lncRNA research in oocyte and embryonic development has increased in recent years, investigations into the effects of vitrification on lncRNA expression remain limited. Specifically, studies addressing how vitrified GV-stage oocytes alter lncRNA expression profiles during porcine oocyte maturation and embryogenesis are still in their infancy. To date, no systematic reports have examined the dynamic changes in lncRNA expression at the MII, 4-cell, and blastocyst stages derived from vitrified porcine GV oocytes, nor have such changes been directly linked to developmental potential. Parthenogenetic embryos can eliminate the influence of sperm and are suitable for assessing oocyte quality [35]. To bridge this gap, the present study comprehensively analyzed the impact of vitrification on lncRNA expression profiles in porcine oocytes and embryos by sequencing lncRNAs at the MII, parthenogenetic 4-cell, and parthenogenetic blastocyst stages obtained from both fresh and vitrified GV oocytes. Furthermore, the study delved into how changes in lncRNA expression correlate with the developmental capacity of oocytes. These findings shed new light on the molecular mechanisms responsible for the diminished developmental ability of porcine oocytes caused by vitrification.

## 2. Materials and Methods

In this research, we sourced all chemicals and reagents from Sigma-Aldrich Chemical Company (St. Louis, MO, USA), unless stated otherwise. We procured tissue culture medium 199 (TCM199) and Dulbecco’s phosphate-buffered saline (DPBS) from Thermo Fisher Scientific (Waltham, MA, USA).

### 2.1. Oocyte Collection and Grouping

Ovaries were harvested from healthy gilts at a nearby abattoir and promptly immersed in a warm saline solution (35–37 °C) fortified with dual antibiotics—penicillin and streptomycin. They were promptly transported to the laboratory and cleansed twice. COCs were suctioned from antral follicles (3–8 mm) using a disposable 20 mL syringe equipped with an 18-gauge needle. They were then double-washed in Tyrode’s lactate-HEPES-polyvinyl alcohol (TLH-PVA) solution [36]. The COCs were examined under a stereomicroscope (Olympus, Tokyo, Japan), and those exhibiting consistent cytoplasm were selected for further experimentation. The entire experiment utilized approximately 800 COCs, with high quality from about 120 ovaries.

### 2.2. Oocyte Vitrification and Warming

In accordance with the established procedure detailed in reference [7], porcine oocytes at the GV stage underwent vitrification and subsequent warming as COCs within a laboratory environment kept at a steady 25 °C. The basic medium (BM) for all solutions consisted of DPBS enhanced with 20% (*v*/*v*) synthetic serum substitute (Irvine Scientific, Santa Ana, CA, USA). During the vitrification process, oocytes were initially washed in BM and then brought to equilibrium in a 5% (*v*/*v*) ethylene glycol (EG) for 10 min at room temperature (RT, 25 °C). Groups of approximately 10–15 oocytes were then transferred into a vitrification solution containing 0.6 M sucrose, 50 mg/mL polyvinylpyrrolidone, and 35% (*v*/*v*) EG, immersed for 20–30 s at RT, and positioned on the tip of a Cryotop carrier (Kitazato Biopharma, Shizuoka, Japan) before being plunged into liquid nitrogen (LN_2_). The entire procedure, from vitrification solution exposure to LN2 immersion, took less than 1 min. For warming, oocytes on the Cryotop were sequentially transferred from LN_2_ to 1.0 M sucrose for 1 min at 42 °C, followed by gradual transfer to 0.5 M and 0.25 M sucrose for 2.5 min each. Finally, the oocytes were washed in BM for 5 min and subjected to in vitro maturation (IVM).

### 2.3. Oocyte IVM

IVM was conducted using 24-well plates (Costar, Corning, NY, USA), with each well housing 500 µL of maturation medium covered with mineral oil. The plate was incubated at 39 °C with 5% CO_2_ in a humidified atmosphere for 42–44 h [37], with each well accommodating roughly 50–70 oocytes. The IVM medium was a blend of TCM199, enriched with 10% (*v*/*v*) porcine follicular fluid, 10 ng/mL epidermal growth factor, 3.05 mM D-glucose, 0.91 mM sodium pyruvate, 0.57 mM cysteine, and 0.5 μg/mL each of follicle-stimulating hormone and luteinizing hormone. Following the oocyte maturation period, cumulus cells were detached from oocytes through repeated pipetting in 0.1% (*w*/*v*) hyaluronidase. Oocytes that had reached the MII stage were identified by the presence of the first polar body and their characteristic uniform, dark cytoplasm.

### 2.4. Vitro Parthenogenetic Activation and Embryo Culture

Following established protocols [38], parthenogenetic activation and subsequent in vitro embryo cultivation were carried out. MII oocytes were equilibrated in activation solution for 30 s and then promptly placed in a 0.5 mm microslide fusion chamber filled with activation medium. The setup was connected to a BLS CF-150/B Cell Fusion Machine (Budapest, Hungary), where the oocytes received a 1.3 kV/cm direct current (DC) pulse lasting 80 μs. Following activation, oocytes were kept in a specialized solution containing porcine zygote medium-3 (PZM-3) supplemented with 5 μg/mL cytochalasin B and 10 μg/mL cycloheximide for 4 h, after which they were moved to PZM-3 medium. Subsequent embryo culture took place at 39 °C with 5% CO_2_ and saturated humidity, with the activation time marked as 0 h. We selectively picked out 4-cell embryos at 48 h, which were free of fragmentation and possessed clear cell borders. Similarly sized blastocysts emerged by 144 h.

### 2.5. Sample Preparation, Library Construction, and RNA Sequencing

Collected MII oocytes were placed in cell lysis buffer, immediately frozen in LN_2_, and stored at −80 °C. Three biological replicates were performed, with each replicate consisting of ten MII oocytes. Total RNA isolation was accomplished using TRIzol reagent (Thermo Fisher, 15596018) with strict adherence to the manufacturer’s recommended protocol. cDNA was synthesized and amplified using the SMART-SeqTM v4 UltraTM Low Input RNA Kit (Takara Bio, Mountain View, CA, USA) in accordance with the manufacturer’s guidelines. Post-amplification, the resulting PCR products underwent purification with Agencourt AMPure XP Microbeads followed by quantification employing a High Sensitivity DNA Kit on a Bioanalyzer (Agilent Technologies, Santa Clara, CA, USA). For library preparation, equivalent quantities of cDNA were processed using Illumina Nextera XT DNA Sample Preparation Kit (Illumina, San Diego, CA, USA), with sequencing subsequently performed on the Illumina NovaSeqTM 6000 platform (Illumina, San Diego, CA, USA) configured for 150 bp paired-end reads (LC-Bio Sciences, Hangzhou, China).

### 2.6. Quality Control and Transcriptome Assembly

The lncRNA data for parthenogenetic 4-cell embryos and blastocysts were sourced from our previous RNA data [39]. To enhance analytical accuracy, we also reanalyzed the 4-cell embryo and blastocyst data starting from the raw data. Raw sequencing reads were processed using Cutadapt (version 2.6) [40] to eliminate adapter contamination, low-quality bases, and ambiguous sequences. For sequence alignment, HISAT2 (version 2.2.1) [41] was employed to align the reads against the Sus scrofa reference genome (Sscrofa11.1) obtained from NCBI. Transcriptome reconstruction was performed using StringTie (version 2.1.6) [42]. Transcripts reconstructed were matched with the reference transcriptome via gffcompare (version 0.9.8) [43] and then categorized into coding or non-coding.

### 2.7. Screening of Candidate LncRNA

lncRNA candidates emerged through location-based analysis, filtering protocols, and coding capacity prediction. The identification process was as follows: (i) transcripts longer than 200 bp were selected; (ii) transcripts overlapping with database-annotated exon regions were removed using Cuffcompare, while lncRNAs already annotated in the database and overlapping with exons of assembled transcripts were retained as known lncRNAs for subsequent analysis; (iii) transcripts belonging to five specific classes (i, j, o, u, x) were extracted using the class_code information from Cuffcompare results, where i indicates transcripts fully contained within a reference intron, j denotes multi-exon transcripts with at least one junction match, o represents transcripts with other same-strand overlaps with reference exons, u indicates unknown intergenic transcripts, and x denotes exon overlaps on the opposite strand of the reference molecule; and (iv) transcript coding potential was assessed, and those with coding potential were excluded. Coding capacity was evaluated using both the coding potential calculator (CPC, version 0.9-r2) and the coding–non-coding index (CNCI, version 2.0) [44,45]. Transcripts identified as non-coding by both methods were retained to establish the final predicted lncRNA dataset. All lncRNAs, whether novel or known, underwent subsequent quantitative assessment.

### 2.8. Analysis of LncRNAs

The DESeq2 package [46] was employed to normalize lncRNA counts across all experimental groups. Quantification of lncRNA expression levels was carried out using the FPKM, which measures fragments per kilobase of exon per million mapped reads. Differential expression analysis between treatment groups pinpointed significantly altered lncRNAs by applying stringent cutoffs: a *q*-value threshold below 0.05 coupled with an absolute log2 fold change exceeding 1.

### 2.9. Target Gene Prediction

To evaluate the biological significance of lncRNAs, we analyzed the roles of their potential targets in both *cis*- and trans-regulation. Protein-coding genes nestled within 100 kb upstream and downstream of each lncRNA were identified to assess possible cis-regulatory functions. Enrichment analyses of gene ontology (GO) terms and Kyoto encyclopedia of genes and genomes (KEGG) pathways were then performed for the identified target genes.

Beyond colocalization, we also investigated genes that were co-expressed with lncRNAs, representing potential trans-regulatory interactions. To assess connections between DELs and differentially expressed genes (DEGs), we performed correlation analysis via the OmicStudio platform (https://www.omicstudio.cn/tool/62, 31 August 2025). We deemed genes as co-expressed with their corresponding lncRNAs if they had a *p*-value under 0.01 and an absolute r-value exceeding 0.95. These matched lncRNA-mRNA pairs were employed to build the core regulatory network, considering both co-expression and colocalization. To further identify key lncRNAs involved in vitrification-induced effects on oocyte maturation and embryo development, we examined the intersection of target genes and DEGs regulated in *cis* or trans. Visualization of the resulting lncRNA-mRNA regulatory networks was performed using Cytoscape (version 3.10.0) [47].

### 2.10. Quantitative Real-Time PCR

Three biological replicates were performed, with each sample consisting of twenty MII oocytes, five parthenogenetic 4-cell embryos, or three parthenogenetic blastocysts. We performed qRT-PCR using the CFX RT-PCR Detection System (Bio-Rad, Hercules, CA, USA). The thermal cycling protocol kicked off with an initial denaturation step at 95 °C for 1 min, followed by 40 cycles consisting of a denaturation phase at 95 °C for 10 s and an annealing/extension phase at 60 °C for 15 s. Each sample was analyzed in triplicate technical replicates, using GAPDH as a benchmark for consistency. The level of gene expression was determined via the 2^−ΔΔCT^ approach, with corresponding primer sequences detailed in Supplementary Table S1.

### 2.11. Statistical Analysis

Univariate analysis of variance was conducted using SPSS 2.0 software. Data are presented as least squares means ± SEM, and differences were considered statistically significant at *p* < 0.05. Graphs were generated using GraphPad Prism 10. The graphical abstract was created by the website biogdp.com. Correlation analysis of the samples revealed strong correlations within the samples, while correlations between different developmental stages were weaker. The correlation between DELs and DEGs was conducted through the OmicStudio platform. Genes satisfying both *p*-values below 0.01 and |r-value| exceeding 0.95 were deemed to exhibit co-expression with their corresponding lncRNAs.

## 3. Results

The data presented in Supplementary Table S1 revealed that survival rates for vitrified GV oocytes post-warming and IVM were 88.6% and 83.9%, respectively, which were notably lower compared to their fresh GV oocytes (100% and 92.2%, respectively). Additionally, the MII rates following IVM were virtually identical for both fresh and vitrified GV oocytes, at 88.9% and 86.7%. Post-parthenogenetic activation, the rates of cleavage and blastocyst formation from vitrified GV oocytes were 75.4% and 26.1%, respectively, substantially lower than those of fresh GV oocytes, which were 88.1% and 55.7%.

### 3.1. Identification and Distribution of lncRNAs

**Figure 1:**
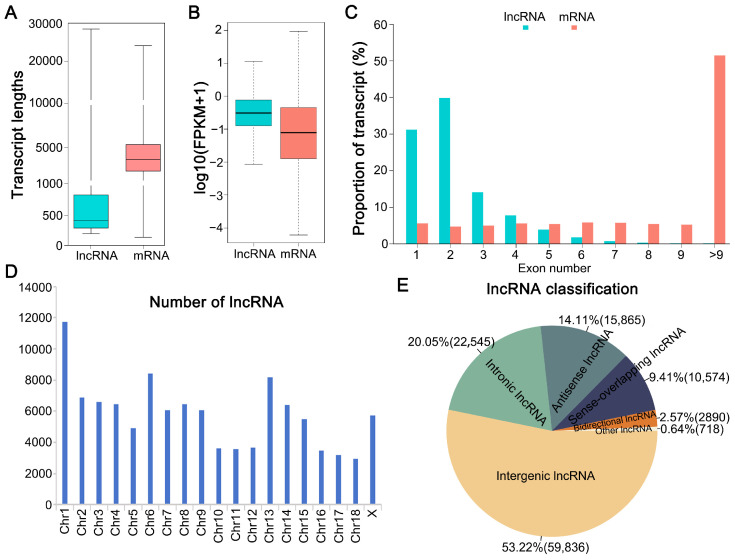
LncRNAs were detected in MII oocytes and parthenogenetic embryos after vitrification at the GV stage: (**A**) LncRNA and mRNA transcript length distributions. (**B**) lncRNA and mRNA expression levels using log10 (FPKM + 1). (**C**) Number of exons in lncRNAs and mRNAs. (**D**) Distributions of lncRNAs in pig chromosomes. (**E**) lncRNA categories.

Following the filtering process and assessment of protein-coding potential in non-coding transcripts derived from clean reads, we pinpointed a substantial pool of 110,141 potential non-coding transcripts. Of this total, 6035 were classified as known lncRNAs, while the remaining 104,106 were novel lncRNAs. When stacked up against mRNA transcripts, we noticed that the length (Figure 1A), expression levels (Figure 1B), and the count of exons (Figure 1C) all alliged with the typical traits of lncRNAs. Chromosome 1 contained the highest number of lncRNAs, followed by chromosomes 6, 13, 2, 3, and 4, whereas chromosome 18 harbored the fewest (Figure 1D). Among the identified lncRNAs, large intergenic lncRNAs were the most abundant, accounting for 53.22% of the total, followed by intronic lncRNAs, which represented 20.53% (Figure 1E).

### 3.2. Differential Expression of LncRNAs in MII Oocytes and Parthenogenetic Embryos Derived from Oocytes Vitrified at the GV Stage

**Figure 2:**
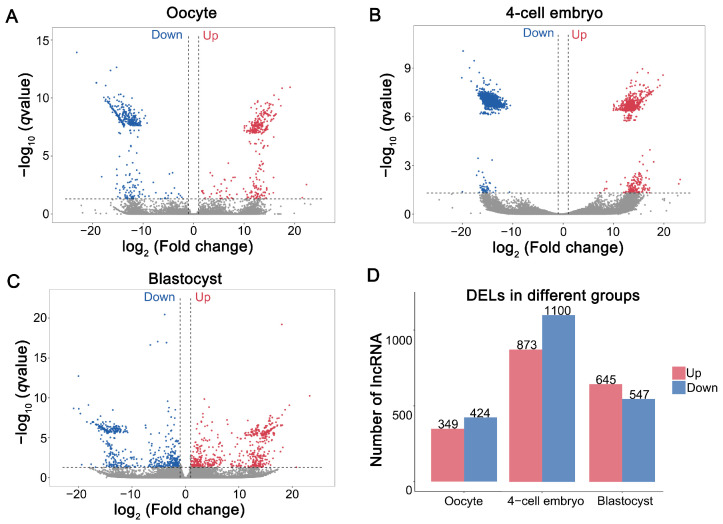
Differential expression of lncRNAs (DELs) in mature oocytes and parthenogenetic embryos produced from vitrified GV oocytes: (**A**) Volcano plots depicting DELs during the mature oocyte stage. (**B**) Volcano plots highlighting DELs at the parthenogenetic 4-cell embryo stage. (**C**) Volcano plots presenting DELs from the parthenogenetic blastocyst stage comparison. “Down” and “Up” indicate “decreased” and “increased”. (**D**) The table details the count of DELs across the mature oocyte, parthenogenetic 4-cell embryo, and parthenogenetic blastocyst comparisons. Red bars represent the upregulated DELs and while blue bars denote the downregulated ones.

Compared with the fresh group, 773 DELs were identified in MII oocytes derived from vitrified GV-stage oocytes after IVM, including 349 upregulated and 424 downregulated DELs (VO vs. FO) (Figure 2A,D, Supplementary Table S2). Following parthenogenetic activation of vitrified GV-stage oocytes matured in vitro, 1973 DELs were detected in parthenogenetic 4-cell embryos, comprising 873 upregulated and 1100 downregulated DELs (V4C vs. F4C) (Figure 2B,D, Supplementary Table S2). In parthenogenetic blastocysts, 1192 DELs were identified, with 645 upregulated and 547 downregulated DELs (VB vs. FB) (Figure 2C,D, Supplementary Table S2). The clustering heatmap confirmed the high reliability of the samples (Figure S1A–C). Notably, only a single DEL overlapped across the MII oocytes, parthenogenetic 4-cell embryos, and parthenogenetic blastocysts (Figure S2). These results indicate that vitrified GV oocytes markedly alter lncRNA expression in mature oocytes and have downstream effects on embryonic development.

### 3.3. QPCR Validation of RNA-Seq Data

**Figure 3:**
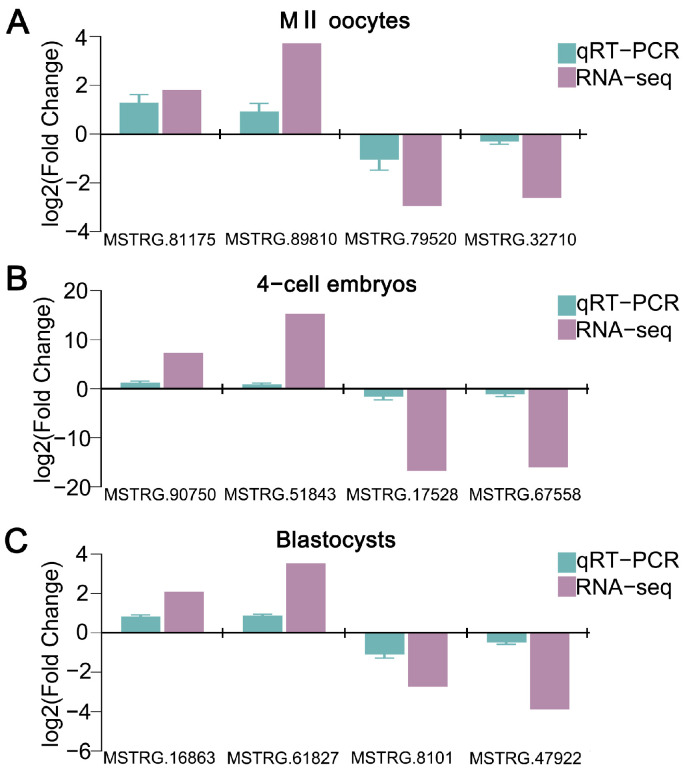
qPCR-based validation of RNA-seq data. qRT-PCR verification of DELs identified in M II oocytes (**A**), parthenogenetic 4-cell embryos (**B**), and parthenogenetic blastocysts (**C**) produced from vitrified GV oocytes. Blue bars indicate qRT-PCR data, and purple bars display RNA-seq results.

To validate the precision and trustworthiness of our RNA sequencing results, four DELs were chosen per group, yielding a total of 12 DELs for qPCR validation (oocytes: MSTRG.81175, MSTRG.89810, MSTRG.79520, MSTRG.32710; 4-cell embryos: MSTRG.90750, MSTRG.51843, MSTRG.17528, MSTRG.67558; blastocysts: MSTRG.16863, MSTRG.61827, MSTRG.8101, MSTRG.47922). The qPCR results demonstrated that all 12 DELs displayed expression patterns consistent with those observed in the RNA-seq (Figure 3A–C), thereby validating our sequencing analysis credibility.

### 3.4. Prediction of Target Genes for DELs in Mature Oocytes Derived from Vitrified GV Oocytes and Enrichment Analysis

**Figure 4:**
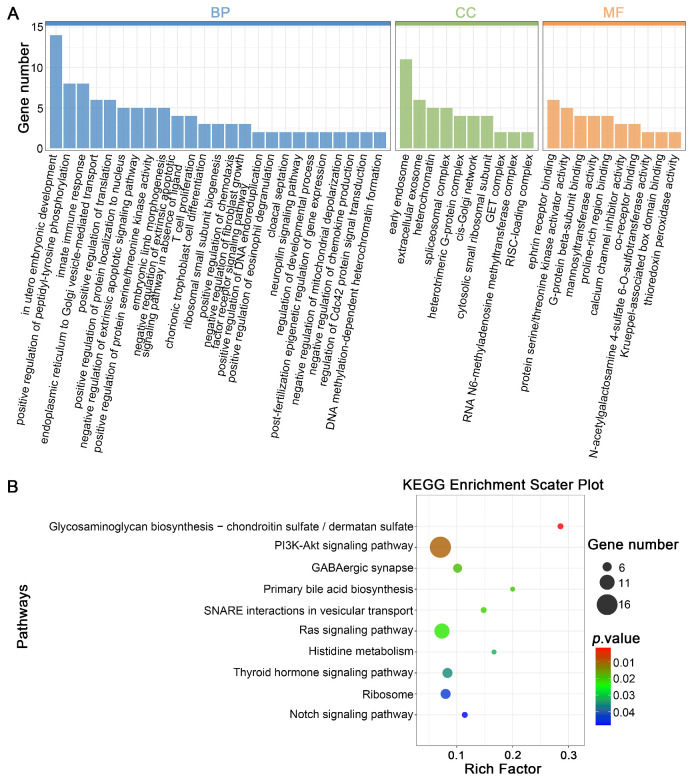
Functional enrichment analysis of *cis*-regulated target genes (cis-genes) by differential expression lncRNAs (DELs) in mature oocytes obtained from vitrified GV oocytes: (**A**) Barplot illustrating GO term enrichment for *cis*-genes by DELs. (**B**) Scatter diagram depicting KEGG pathway enrichment for *cis*-genes by DELs. The enrichment metric reflects the proportion of DEL-associated cis-genes mapped to specific pathways relative to the total population of lncRNA-regulated *cis*-genes in the reference genomic dataset. Dot size represents DEL-associated *cis*-gene quantity, while dot color denotes *p*-value ranges.

To investigate the potential function of the DELs pinpointed in our research, we analyzed their anticipated *cis*- and trans-regulatory targets. For *cis*-regulation, protein-coding genes situated within 100 kilobases upstream or downstream of the DELs were identified, resulting in 773 DELs associated with 1113 protein-coding genes (Supplementary Table S3). Taking this analysis a step further, we subjected these *cis*-target genes to GO and KEGG enrichment analyses to get a better handle on their functions. The enriched GO terms were then grouped into three main categories: biological process (BP), cellular component (CC), and molecular function (MF). In total, 429 GO terms were significantly enriched, including 314 BP, 26 CC, and 89 MF terms (Figure 4A, Supplementary Table S4). The most enriched BP terms were mainly related to “chorionic trophoblast cell differentiation”, “positive regulation of peptidyl-tyrosine phosphorylation”, “negative regulation of extrinsic apoptotic signaling pathway”, “regulation of developmental process”, “post-fertilization epigenetic regulation of gene expression”, and “in utero embryonic development”. Moreover, KEGG pathway analysis disclosed significant enrichment in various routes such as “glycosaminoglycan biosynthesis”, “PI3K-Akt signaling pathway”, “Ras signaling pathway”, and “Notch signaling pathway” (Figure 4B, Supplementary Table S4). Collectively, these results indicate that the identified lncRNAs may regulate oocyte development by modulating nearby protein-coding genes via cis-regulation.

**Figure 5:**
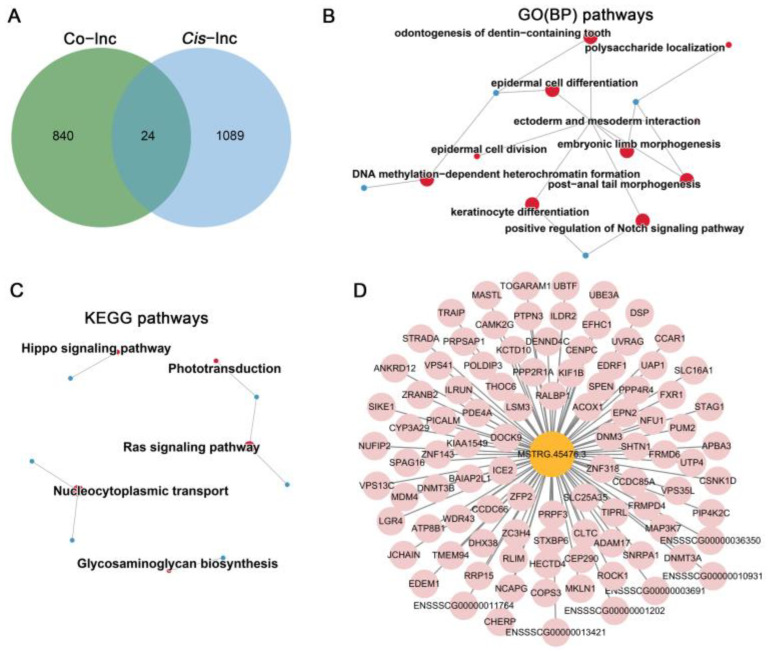
Characterization of key lncRNA-mRNA networks in mature oocytes originating from vitrified GV oocytes: (**A**) *Cis*-regulation Venn diagram illustrating co-expressed genes; common regions refer to potential central genes. (**B**) GO enrichment study of candidate core genes targeted by core lncRNAs. (**C**) KEGG enrichment analysis for core lncRNA-targeted genes. The red circles represent the enriched terms, while the blue circles represent the candidate core genes targeted by core lncRNAs. The diameter of the circles indicates the number of central genes. (**D**) Regulatory network between the core lncRNA MSTRG.45476.3 and its target genes, with pink symbols designating positively regulated genes.

The potential trans-regulatory functions of these DELs were assessed through expression correlation analysis, which identified 48,648 predicted interactions between 759 DELs and 864 DEGs (Supplementary Table S5). To pinpoint key lncRNAs involved in vitrification-induced oocyte maturation, a trans-regulatory lncRNA-mRNA interaction network was constructed using the DELs and their predicted DEG targets. Our analysis revealed that 24 *cis*-genes overlapped with co-expressed genes (Figure 5A, Supplementary Table S6), corresponding to 21 core lncRNAs. GO and KEGG enrichment studies were then conducted to explore the potential functions of these *cis*-genes. Predominant GO BP enrichments were mainly associated with “DNA methylation-dependent heterochromatin formation”, “epidermal cell differentiation”, “positive regulation of Notch signaling pathway”, “embryonic limb morphogenesis”, and “ectoderm and mesoderm interaction” (Figure 5B, Supplementary Table S7). KEGG pathway analysis further showed significant enrichment in “nucleocytoplasmic transport”, “glycosaminoglycan biosynthesis”, “Hippo signaling pathway”, and “Ras signaling pathway” (Figure 5C, Supplementary Table S7). Notably, within the co-expression network, one lncRNA (MSTRG.45476.3) was found to regulate the majority of mRNAs in the network (Figure 5D).

### 3.5. Prediction of Target Genes for DELs in Parthenogenetic 4-Cell Embryos Derived from Vitrified GV Stage Oo-Cytes and Enrichment Analysis

**Figure 6:**
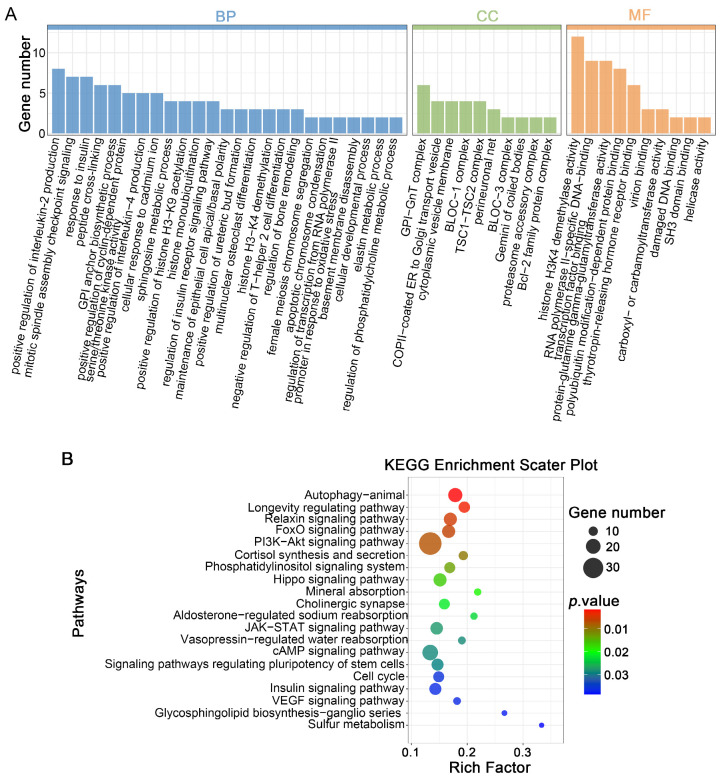
Functional enrichment analysis of *cis*-genes by DELs in parthenogenetic 4-cell embryo originating from vitrified GV oocytes: (**A**) Barplot illustrating GO term enrichment of *cis*-genes by DELs. (**B**) Scatter diagram depicting KEGG pathway enrichment of *cis*-genes by DELs. The enrichment factor is calculated as the proportion of *cis*-genes identified by DELs that are found within a specific pathway relative to the total number of cis-genes from all lncRNAs in our reference gene collection. Dot size represents DELs-associated *cis*-gene quantity, while dot color denotes *p*-value ranges.

In parthenogenetic 4-cell embryos, 1973 DELs were predicted to regulate 2619 target genes through *cis*-regulatory mechanisms (Supplementary Table S3). These *cis*-genes were substantially enriched in 246 GO terms, including 177 BPs, 26 CC, and 43 MF (Figure 6A, Supplementary Table S4). The spotlight fell squarely on BP terms were primarily related to “mitotic spindle assembly checkpoint signaling”, “sphingosine metabolic process”, “positive regulation of cyclin-dependent protein serine/threonine kinase activity”, “positive regulation of histone H3-K9 acetylation”, “histone monoubiquitination”, “histone H3-K4 demethylation”, “female meiosis chromosome segregation”, and “cellular developmental process”. KEGG analysis additionally showed marked overrepresentation in “FoxO signaling pathway”, “PI3K-Akt signaling pathway”, “phosphatidylinositol signaling system”, “Hippo signaling pathway”, “JAK-STAT signaling pathway”, “cell cycle”, and “VEGF signaling pathway” (Figure 6B and Supplementary Table S4). Collectively, these results indicate that lncRNAs affected by vitrification may control embryonic development by adjacent *cis*-regulating genes.

**Figure 7:**
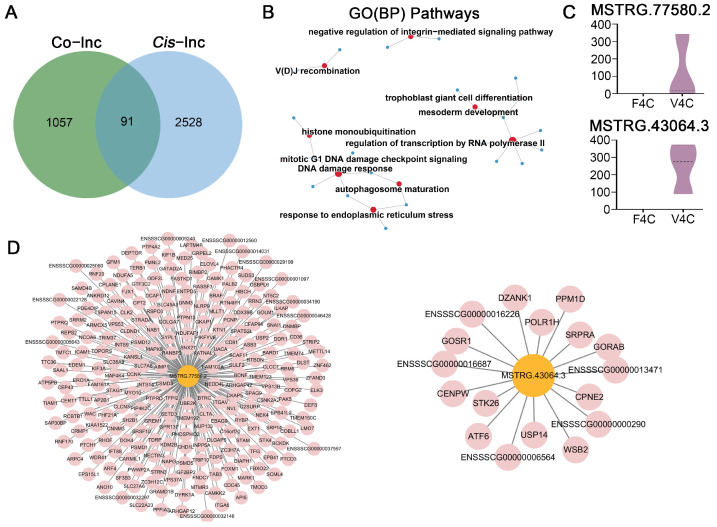
Characterization of key lncRNA-mRNA networks for parthenogenetic 4-cell embryo originating from vitrified GV oocytes: (**A**) *Cis*-regulation Venn diagram illustrating co-expressed genes; common regions refer to potential core genes. (**B**) GO enrichment study of candidate central genes regulated by key lncRNAs. The red circles represent enriched terms, while the blue circles represent the candidate core genes targeted by core lncRNAs. Circle dimensions reflect the quantity of core genes. (**C**) Expression of core lncRNAs for parthenogenetic 4-cell embryo obtained from fresh and vitrified GV oocytes. (**D**) Regulatory network linking core lncRNA MSTRG.77580.2 or MSTRG.43064.3 to their target genes, with pink symbols designating positively regulated genes.

In the current investigation, a robust set of 319,689 lncRNA-mRNA pairings was identified, which included 1148 DEGs and 1972 DELs (Figure 7A, Supplementary Table S5). We then delved deeper into the core lncRNA-mRNA regulatory network based on overlapping targets from both *cis*- and trans-regulation. Our analysis revealed 91 *cis*-genes that had common ground with co-expressed genes, corresponding to 92 core lncRNAs (Supplementary Table S6). Significantly, these *cis*-genes demonstrated substantial enrichment across 271 GO terms, comprising 180 BP, 40 CC, and 51 MF (Figure 7B and Supplementary Table S7). The BP terms that were enriched primarily revolved around “histone monoubiquitination”, “regulation of transcription by RNA polymerase II”, “mesoderm development”, “response to endoplasmic reticulum stress”, “DNA damage response”, and “mitotic G1 DNA damage checkpoint signaling”. KEGG pathway analysis of these cis-genes indicated a single substantially enriched pathway (Supplementary Table S7). Within the co-expression network, one lncRNA (MSTRG.77580.2) was found to regulate the majority of mRNAs (Figure 7C,D). Additionally, we identified a paired lncRNA-mRNA that was detected by both *cis*- and trans-regulatory methods, with MSTRG.43064.3 emerging as a candidate core lncRNA regulating a total of 17 genes (Figure 7C,D).

### 3.6. Prediction of Target Genes for DELs in Parthenogenetic Blastocysts Derived from Vitrified GV Stage Oocytes and Enrichment Analysis

**Figure 8:**
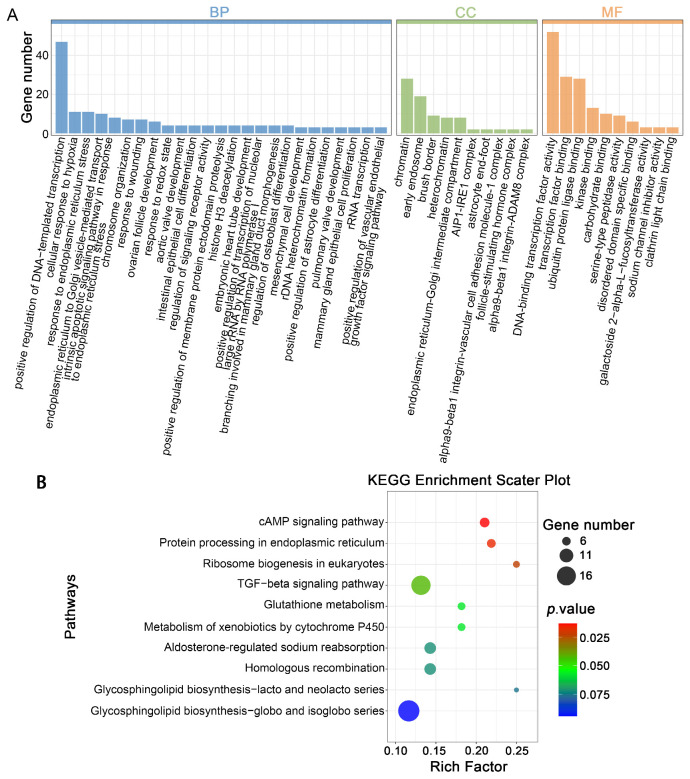
Functional enrichment analysis of *cis*-genes by DELs in parthenogenetic blastocysts obtained from vitrified GV oocytes: (**A**) Barplot illustrating GO term enrichment of cis-genes by DELs. (**B**) Scatter diagram depicting KEGG pathway enrichment of *cis*-genes by DELs. The ‘rich factor’ here refers to the proportion of *cis*-genes by DELs that are enriched in a particular pathway relative to the total number of cis-genes by all lncRNAs within the reference gene set. Dot size represents DELs-associated *cis*-gene quantity, while dot color denotes *p*-value ranges.

In blastocysts, 1192 DELs were identified as regulating 2507 target genes through *cis*-regulatory mechanisms (Supplementary Table S3). The analysis revealed a significant enrichment of *cis*-genes across 297 GO terms, including 199 BPs, 41 CC, and 57 MF (Figure 8A, Supplementary Table S4). The enriched BP terms were mainly related to stress responses, such as “intrinsic apoptotic signaling pathway in response to endoplasmic reticulum stress”, “cellular response to hypoxia”, and “response to endoplasmic reticulum stress”. Additionally, terms associated with developmental and organizational processes, including “mesenchymal cell development”, “chromosome organization”, and “ovarian follicle development”, were also enriched. KEGG pathway analysis further revealed substantial enrichment in pathways including “glutathione metabolism”, “metabolism of xenobiotics by cytochrome P450”, “glycosphingolipid biosynthesis”, and “protein processing in endoplasmic reticulum” (Figure 8B, Supplementary Table S4).

**Figure 9:**
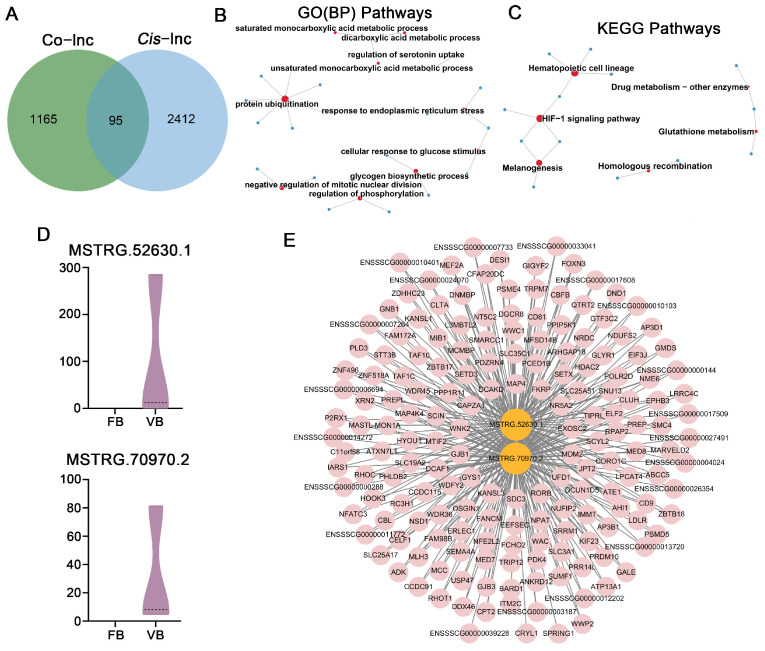
Characterization of key lncRNA-mRNA networks for parthenogenetic blastocyst originating from vitrified GV oocytes: (**A**) *Cis*-regulation venn diagram illustrating co-expressed genes; common regions refer to potential central genes. (**B**) GO enrichment study of candidate core genes targeted by core lncRNAs. (**C**) KEGG enrichment analysis for core lncRNA-targeted genes. The red circles represent the enriched terms, while the blue circles represent the candidate core genes targeted by core lncRNAs. Circle dimensions indicate the number of core genes. (**D**) Expression of core lncRNAs for blastocyst obtaining from fresh and vitrified GV oocytes. (**E**) Core lncRNA-mRNA network of core lncRNAs and their targeted genes; the pink symbol marks a positive regulatory gene.

Furthermore, we delved into the putative trans-regulatory functions of these lncRNAs by leveraging their expression correlation coefficients, which ultimately enabled us to pinpoint 87,589 potential interactions connecting 1192 lncRNAs with 1260 protein-coding genes (Figure 9A, Supplementary Table S5). We took the analysis a step further by consolidating the core lncRNA-mRNA regulatory network through the integration of shared targets that were subject to both *cis*- and trans-regulatory mechanisms. This approach uncovered 95 *cis*-genes that coincided with co-expressed genes, belonging to 93 core lncRNAs (Supplementary Table S6). These *cis*-genes showed notable enrichment across 295 GO terms, including 180 BPs, 44 CC, and 71 MF (Figure 9B, Supplementary Table S7). The enriched BP was primarily related to “protein ubiquitination”, “negative regulation of mitotic nuclear division”, “regulation of phosphorylation”, “glycogen biosynthetic process”, “cellular response to glucose stimulus”, and “response to endoplasmic reticulum stress”. KEGG analysis further showed notable pathway enrichment in the “HIF-1 signaling pathway”, “homologous recombination”, and “glutathione metabolism” (Figure 9C, Supplementary Table S7). Within the co-expression network, two paired lncRNA-mRNA interactions were detected by both regulatory approaches, namely the upregulated lncRNAs MSTRG.52630.1 and MSTRG.70970.2 (Figure 9D), which were also found to modulate the majority of mRNAs in the network (Figure 9E).

## 4. Discussion

Earlier research documented that vitrified porcine oocytes at the GV stage exhibit reduced developmental competence, and several attempts have been made to increase blastocyst yield [16,48,49]. However, vitrification-induced damage can impair both oocytes and their subsequent development. lncRNAs serve as crucial regulatory agents in a wide array of cellular mechanisms. Our investigation focused on DELs across three distinct developmental phases, namely MII oocytes, parthenogenetic 4-cell embryos, and parthenogenetic blastocysts, all originating from both vitrified and fresh GV oocytes. It is well-established that lncRNAs exert trans-regulatory control over oocyte maturation and embryonic progression [19,25] and influence meiosis via chromatin modification through *cis*-regulation [50]. Thus, predicting *cis*- and trans-regulatory interactions of lncRNAs is crucial for understanding their impact on oocyte and embryo development, which is largely determined by their target genes. Here, we constructed a core lncRNA-mRNA network through combined cis- and trans-regulation analysis. This research sheds new light on how vitrification influences the developmental capacity of oocytes, sharpens our understanding of the regulatory framework governing porcine oocyte maturation, and lays the groundwork for creating innovative cryoprotective agents and refining cryopreservation techniques.

Prior research has consistently demonstrated that lncRNA genes tend to be more compact than their protein-coding counterparts, featuring shorter gene lengths, abbreviated transcripts, and fewer exons [23,51,52]. Our findings align with this established pattern, as the lncRNAs we identified followed suit with these distinctive traits. Earlier research has also indicated that mRNA and protein levels in immature oocytes may partially recover during maturation after vitrification and thawing [53,54,55]. Therefore, assessing lncRNA expression during the MII stage provides a more accurate reflection of the effects of vitrification on the subsequent developmental potential of oocytes.

In our research, we identified 773 DELs within MII oocytes sourced from vitrified GV oocytes after IVM, including 349 upregulated and 424 downregulated DELs. GO enrichment analysis of their corresponding target genes brought to light several biological terms intimately connected to the oocyte’s developmental capacity, such as “regulation of developmental process”, “post-fertilization epigenetic regulation of gene expression”, and “in utero embryonic development”. Moreover, KEGG pathway analysis indicated that vitrification may impair oocyte maturation through pathways critical for meiosis and cell cycle regulation, including the PI3K-Akt, Ras, and Notch signaling pathways [56,57,58].

To identify key core lncRNAs, we integrated *cis*- and trans-regulatory predictions and identified 21 core lncRNAs corresponding to 24 DEGs. GO and KEGG analyses of these genes also highlighted the Ras and Notch signaling pathways. Among these, *YAP1* was downregulated, while *TP63* and *NSMCE4A* were upregulated in vitrified MII oocytes. YAP1 is demonstrated to modulate cellular proliferation, differentiation, survival, and oocyte maturation; its activation enhances IVM and subsequent embryonic development [59]. In contrast, increased TP63 expression can induce downstream targets that trigger oocyte apoptosis [60]. NSMCE4A, a critical component of the SMC5/6 complex, is involved in DNA damage repair [61]; its upregulation suggests vitrification-induced DNA damage during meiosis. These genes are regulated by lncRNAs MSTRG.87298, MSTRG.27232.6, and MSTRG.34876.1, respectively, indicating the pivotal roles of these lncRNAs in oocyte maturation.

Furthermore, the latest research has flagged unusual epigenetic alterations in vitrified oocytes and embryos [62,63]. Consistently, our GO enrichment analysis identified significant terms tied to epigenetic changes, including “DNA methylation-dependent heterochromatin formation”, “arginyl-tRNA-protein transferase activity”, and “protein arginylation”. These findings suggest that vitrification not only affects oocyte maturation but also compromises their developmental competence. Compared with embryos, however, vitrified GV oocytes appear to undergo relatively smaller lncRNA expression changes during maturation.

This study demonstrates that 1973 lncRNAs are differentially expressed at the parthenogenetic 4-cell stage sourced from vitrified GV oocytes, representing the largest group observed during oocyte maturation and across the two embryonic developmental stages. GO enrichment of coregulated targets by these DELs in both *cis*- and trans-acting manners revealed significant associations with protein-related processes, including “histone monoubiquitination”, “polyubiquitin modification-dependent protein binding”, “histone H2B conserved C-terminal lysine ubiquitination”, “ubiquitin-dependent protein catabolic process”, “histone H3K36 methyltransferase activity”, and “histone H4 deacetylation”. These findings suggest that vitrified pig GV oocytes may influence ZGA at the 4-cell stage. Furthermore, vitrification of both oocytes and embryos is known to trigger stress-related cellular responses [64]. In line with this, we detected multiple GO terms associated with vitrification-induced stress reactions, including “DNA damage response”, “response to endoplasmic reticulum stress”, “mitotic G1 DNA damage checkpoint signaling”, “regulation of oxidative stress-induced intrinsic apoptotic signaling pathway”, and “positive regulation of oxidative phosphorylation”. Previous studies have reported that endoplasmic reticulum stress negatively affects mammalian oocyte maturation and embryonic development [65], and is present in vitrified oocytes and embryos [66,67]. Additionally, our GO analysis highlighted enrichment in pathways associated with cell differentiation, development, and metabolism, including “trophoblast giant cell differentiation”, “mesoderm development”, “B cell homeostatic proliferation”, “receptor metabolic process”, “GDP-L-fucose metabolic process”, and “NADH metabolic process”. Alterations in the genes within these pathways may further impact subsequent embryonic development. Moreover, KEGG pathway analysis revealed that the target genes regulated by *cis*-acting elements of DELs were markedly enriched in multiple pathways playing pivotal roles in oocyte maturation and embryonic development, such as the “FoxO signaling pathway”, “PI3K-Akt signaling pathway”, “Hippo signaling pathway”, “JAK-STAT signaling pathway”, and “VEGF signaling pathway”. The MZT initiation time of pig embryos occurs at the 4-cell stage. At this stage, the embryo needs to complete the critical transition from being dependent on maternal reserves to autonomous genomic regulation. As lncRNAs are important regulators of gene expression, they undertake the dual tasks of eliminating maternal lncRNAs and activating zygotic lncRNAs during this process, directly leading to a rise in lncRNA quantity. The vitrification of GV oocytes may exacerbate this process disorder. The above data indicate that vitrification causes abnormalities in epigenetic modifications and DNA damage repair, and these factors may result in a considerable rise in the number of DELs at the 4-cell stage.

1192 lncRNAs exhibited differential expression in parthenogenetic blastocysts from vitrified GV oocytes. Some research has found that parthenogenetically activated embryos are more sensitive to environmental stresses compared to normally fertilized embryos [68,69]. Our GO results indicate that not only at the parthenogenetic 4-cell stage, but also at the parthenogenetic blastocyst stage, embryos are enriched for terms related to cellular stress, such as “response to endoplasmic reticulum stress” and “cellular response to glucose stimulus”. Besides, vitrification was shown to affect GO terms including “negative regulation of mitotic nuclear division”, “negative regulation of germinal center formation”, and “negative regulation of T-helper cell differentiation”, all of which are linked to blastocyst trophectoderm and inner cell mass formation. Notably, genes enriched within these categories were consistently downregulated, suggesting that such downregulation may contribute to a delayed formation process in parthenogenetic blastocysts from vitrified GV oocytes. KEGG enrichment analysis further highlighted two key pathways: the HIF-1 signaling pathway and glutathione metabolism. Prior research has shown the crucial role of the HIF-1 signaling pathway in regulating oocyte maturation and early embryonic development [70], while restoration of glutathione during recovery culture after thawing can mitigate the detrimental effects of vitrified oocytes and protect cells from oxidative stress damage [71]. Consistent with this, our data revealed that genes enriched in both pathways, including *EIF4E*, *TFRC*, *CREBBP*, *GSTA1*, and *LAP3*, were all downregulated. Therefore, we propose that suppression of the “HIF-1 signaling pathway” and “glutathione metabolism” may contribute to the abnormal development observed in parthenogenetic blastocysts obtained from vitrified GV oocytes.

Furthermore, we analyzed the commonly occurring DELs across the three groups and observed that lncRNA MSTRG.55131.4 was consistently downregulated in MII oocytes, parthenogenetic 4-cell embryos, and parthenogenetic blastocysts sourced from vitrified GV oocytes. Examination of the target genes regulated by MSTRG.55131.4 revealed that ADNP homeobox 2 (*ADNP2*) was a shared target in MII oocytes and 4-cell embryos, where it was significantly downregulated in samples derived from vitrified GV oocytes. Similarly, cytochrome B5 type A (*CYB5A*) and RE1 silencing transcription factor (*REST*) were identified as common targets in 4-cell embryos and blastocysts, and both genes were likewise downregulated in these developmental stages following vitrification of GV oocytes. Prior studies have demonstrated that ADNP2 functions as a transcription factor broadly expressed during early vertebrate embryonic development and is closely associated with oxidative stress [72]; CYB5A is integral to the cholesterol biosynthesis pathway in lipid metabolism [73]; and REST, an inhibitor of the TGFβ signaling pathway in pigs, plays a crucial role in nuclear transfer-mediated reprogramming [74]. Taken together, the downregulation of these genes indicates that vitrified GV oocytes induce oxidative stress and lipid metabolic disturbances, ultimately contributing to abnormal embryonic development.

In addition, we constructed an lncRNA-mRNA regulatory network based on DELs and their *cis*- and trans-regulated DEGs. In mature oocytes, 21 core lncRNAs were identified, with lncRNA MSTRG.45476.3 regulating the majority of mRNAs in the network. At the parthenogenetic 4-cell stage, 92 core lncRNAs were detected, among which lncRNA MSTRG.77580.2 exerted the greatest regulatory influence. Notably, a pair of lncRNA-mRNA interactions (MSTRG.43064.3-DZANK1) was identified by both cis and trans analyses. In parthenogenetic blastocysts, 93 core lncRNAs were discovered, including two lncRNA-mRNA pairs (MSTRG.52630.1-DDX46 and MSTRG.70970.2-FCGRT), both of which also regulated the majority of network-associated mRNAs. Importantly, four core lncRNAs (MSTRG.45476.3, MSTRG.77580.2, MSTRG.52630.1, and MSTRG.70970.2) shared a common target gene, ankyrin repeat domain 12 (*ANKRD12*). Previous research has shown that circANKRD12 expression is upregulated in endothelial cells under oxidative stress [75]. Consistent with this, our findings demonstrated that ANKRD12 expression increased in MII oocytes sourced from vitrified GV oocytes, yet was downregulated at the parthenogenetic 4-cell and blastocyst stages. This pattern suggests that vitrified GV oocytes may partially repair freezing-induced damage during the maturation process, but such repair is insufficient to sustain later embryonic development.

## 5. Conclusions

In summary, this research evaluated variations in lncRNA levels among MII oocytes, parthenogenetic 4-cell embryos, and parthenogenetic blastocysts sourced from vitrified and fresh GV oocytes, thereby revealing potential mechanisms by which vitrification influences oocyte and embryonic development. These findings provide valuable insights for advancing our understanding of embryonic developmental processes of the vitrified oocytes. Further studies may explore strategies to regulate lncRNA expression, with the goal of enhancing the developmental competence of vitrified oocytes and embryos.

## Data Availability

The authors declare that the data supporting the findings of this study are available within the paper and its Supplementary Materials or are available from the corresponding author upon reasonable request.

## Supplementary Materials

The following supporting information can be downloaded at: https://www.mdpi.com/article/10.3390/cells14221808/s1, Figure S1: Cluster heatmap analysis of DELs in mature oocytes and embryos derived from vitrified porcine GV oocytes; Figure S2: Venn diagrams of differentially expressed lncRNAs in mature oocytes and embryos derived from vitrified porcine GV oocytes; Table S1: qPCR primer and overview of survival, nuclear maturation and embryonic development; Table S2: Total_lncRNA_differential_expression in different group; Table S3: lncRNA *cis*-regulatory target genes; Table S4: GO and KEGG enrichment analysis of the cis-regulated target genes by DELs; Table S5: lncRNA trans-regulatory genes; Table S6: Core lncRNA-mRNA; Table S7: GO and KEGG enrichment analysis of target genes by core DELs.

## Abbreviations

The following abbreviations are used in this manuscript:

IVM	In vitro maturation
LncRNAs	Long non-coding RNAs
GV	Germinal vesicle
MII	Metaphase II
DELs	Differentially expressed lncRNAs
COCs	Cumulus–oocyte complexes
MZT	Maternal-to-zygotic transition
ZGA	Zygotic genome activation
PZM-3	Porcine zygote medium-3
GO	Gene ontology
KEGG	Kyoto encyclopedia of genes and genomes
DEGs	Differentially expressed genes
