# Supplementary material for: Long Non-Coding RNA Analysis of Vitrified Porcine Immature Oocytes During Maturation and Early Parthenogenetic Embryo Development
Source: Cells. 2025 Nov 18;14(22):1808. doi: 10.3390/cells14221808 (PMC12651464; doi:10.3390/cells14221808)
Supplement: Supplementary file 1 [file cells-14-01808-s001.zip › Supplementary figures.pdf]

Supplementary Figures

Long Non-Coding RNA Analysis of Vitrified Porcine Immature Oocytes During Maturation and Early Parthenogenetic Embryo Development

De-Cai Xiang [1,†], Zhen He [1,†], Shi-Qi Pu [1], De-Meng Mu [1], Jing Fu [1], Wen-Juan Chen [1], Jun-Yu Jiang [2], Xue-Mei Li [2], Bao-Yu Jia [2,*] and Guo-Quan Wu [1,*]

[1] National Regional Genebank (Yunnan) of Livestock and Poultry Genetic Resources, Yunnan Provincial Engineering Laboratory of Animal Genetic Resource Conservation and Germplasm Enhancement, Yunnan Animal Science and Veterinary Institute, Kunming 650224, China; askalm@163.com (D.-C.X.); 18463287165@163.com (Z.H.); psqshiqi@163.com (S.-Q.P.); Mudemeng1219@163.com (D.-M.M.); fj11260027@163.com (J.F.); chenwenjuanly006@163.com (W.-J.C.)

[2] Key Laboratory for Porcine Gene Editing and Xenotransplantation in Yunnan Province, College of Veterinary Medicine, Yunnan Agricultural University, Kunming 650201, China; 13987988411@163.com (J.-Y.J.); lxm19187386050@163.com (X.-M.L.)

* Correspondence: jiabaoyu2009@163.com (B.-Y.J.); wuguoquan1982@163.com (G.-Q.W.)

† These authors contributed equally to this work.

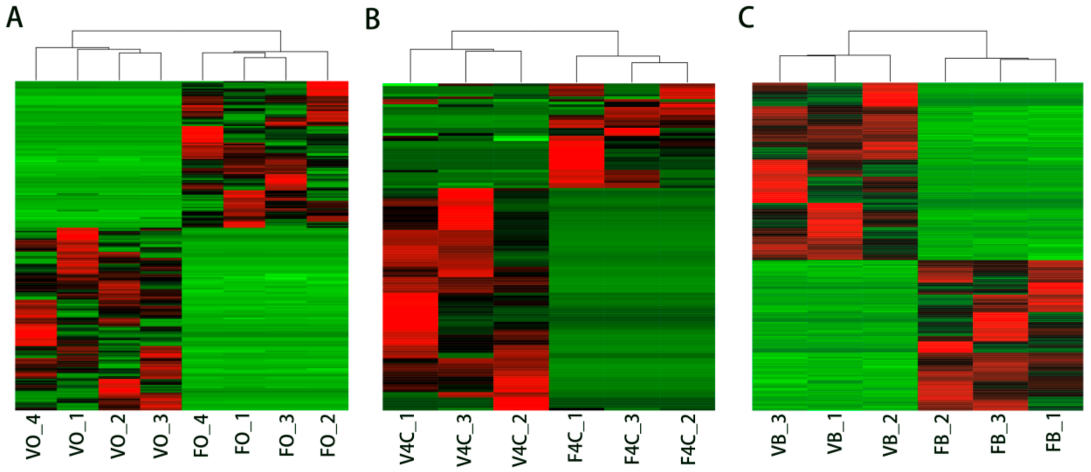

Figure S1. Cluster heatmap analysis of DELs in mature oocytes and embryos derived from vitrified porcine GV oocytes: (A) Hierarchical clustering analysis of DELs in mature oocytes derived from vitrified and fresh porcine GV oocytes (VO vs FO). Red denotes upregulation while green denotes downregulation. 1, 2, 3 and 4 represent sample replicates for each group. (B) Hierarchical clustering analysis of DELs in 4-cell embryo derived from vitrified and fresh porcine GV oocytes (V4C vs F4C). (C) Hierarchical clustering analysis of DELs in blastocyst derived from vitrified and fresh porcine GV oocytes (VB vs FB). Red denotes upregulation while green denotes downregulation. 1, 2 and 3 represent sample replicates for each group.

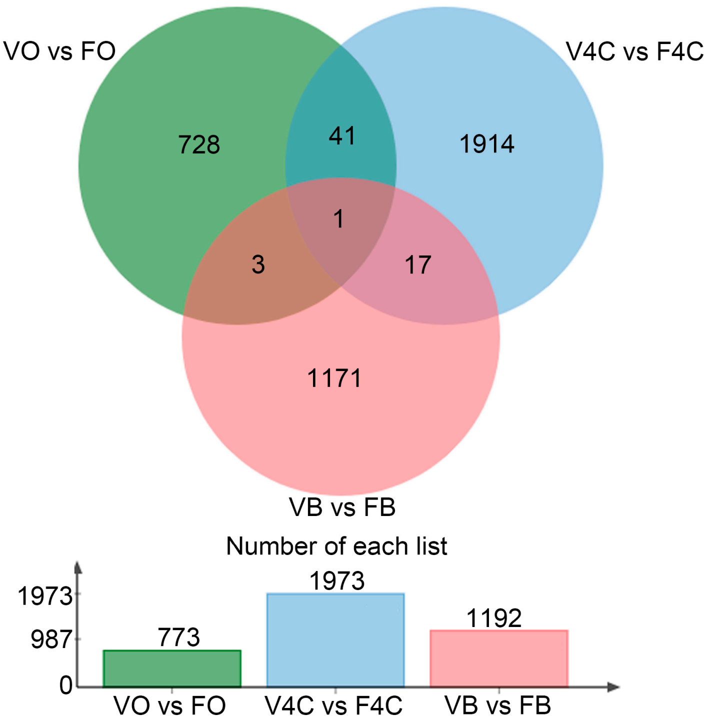

Figure S2. Venn diagrams of differentially expressed lncRNAs in mature oocytes and embryos derived from vitrified porcine GV oocytes.
