# Supplementary material for: Long Non-Coding RNA Analysis of Vitrified Porcine Immature Oocytes During Maturation and Early Parthenogenetic Embryo Development
Source: Cells. 2025 Nov 18;14(22):1808. doi: 10.3390/cells14221808 (PMC12651464; doi:10.3390/cells14221808)
Supplement: Supplementary file 1 [file cells-14-01808-s001.zip › Supplementary Table S1 qPCR primer and overview of survival, nuclear maturation and embryonic development.pdf]

Primers(5′-3′) used in this study.

lncRNA		Primer sequence(5′-3′)
MSTRG.81175	F	TGAACCGGGAGCTAAGAAGG
	R	CTTTGGGCACAGGTCTCTCT
MSTRG.89810	F	GCACATGGTAGGTTGTCAGC
	R	GCACATGGTAGGTTGTCAGC
MSTRG.79520	F	ACTTGGGGCATATGGAGGTT
	R	TGAAGGTCACAGATGCCACT
MSTRG.32710	F	GGAAATGAGGGCCACAACTG
	R	TTGGACCCTACCTTGTTGCT
MSTRG.90750	F	ACGCTCTGCAACCAAAATGT
	R	ACGTGCGCTGAGTTTGTTTA
MSTRG.51843	F	GCCACCTACTGTCACTTTGC
	R	TAGTGCCACAGATGTCCCAG
MSTRG.17528	F	CCCGCATCTCAAAGGTATGG
	R	GCCATCAGAGAAAACGCACA
MSTRG.67558	F	ACCTTTCAGCCTCCACTCTC
	R	AGAGAACGGGGATGTGTTGT
MSTRG.16863	F	TTCTTTGCTCCATGAACGGC
	R	AGTGGCTTAAGGATGCAGGT
MSTRG.61827	F	GCGCAGTGGTTAACGAATCC
	R	CAACCTACACCACAGCTCAC
MSTRG.8101	F	TTGCGTTGTTGTGGCTCTAG
	R	TGGCACACAAAAGTTCCCAG
MSTRG.47922	F	TTTGCTATTTCTTGGGCCGC
	R	TTGCGTTGTTGTGGCTCTAG

Overview of survival and nuclear maturation of fresh and vitrified porcine immature oocytes.

Groups	No. of oocytes	Survival rate after warming (%)	Survival rate after IVM (%)	Nuclear maturation rate (%)
Fresh	320	100.0 ± 0^{a}	92.2 ± 1.2^{a}	88.9 ± 1.4
Vitrified	455	88.6 ± 1.3^{b}	83.9 ± 1.6^{b}	86.7 ± 1.5

Data are expressed as mean ± SEM values.

^{a,b}Different superscripts in the same column indicate significant differences ($P < 0.05$).

Overview of developmental competence of fresh and vitrified porcine immature oocytes after parthenogenetic activation.

Groups	No. of oocytes	Cleavage rate (%)	Blastocyst rate (%)
Fresh	151	88.1 ± 1.2^{a}	55.7 ± 1.5^{a}
Vitrified	183	75.4 ± 1.9^{b}	26.1 ± 2.7^{b}

Data are expressed as mean ± SEM values.

^{a,b}Different superscripts in the same column indicate significant differences ($P < 0.05$).
